# Scirrhus of Duodenum, Pylorus, and Head of Pancreas

**Published:** 1866-10

**Authors:** E. Allen Wood

**Affiliations:** McKeesport, Pa.


*Scirrhus of Duodenum, Pylorus, and Head of Pancreas.* By E. Allen Wood, M. D., McKeesport, Pa.

The history of the following case is remarkable. The unnatural phenomena detailed are well calculated to create doubts of their genuineness. The author would not have ventured to publish it, were it not for the daily notes left of the case, and from which the following is taken; and more especially since he has the concurment of Dr. Wm. B. Lank, a highly respectable physician, who daily watched with him the progress and termination of the case, and who, with Drs. Donaldson and Miller, saw the structural change at the post mortem examination.

Mr. John S., aged 50, farmer; hard worker and very great eater. Has had dyspepsia for many years, and has been subject to severe attacks of bilious colic. Bowels have been very torpid for a long time, more especially for the last three years have they been obstinately constipated. Five months ago, had one of his attacks of colic, which was more than usually severe. Ever since that time he has been troubled with pain in right hypochondrium, and cannot lie with comfort on his right side. For five months, has had no full and free evacuation from his bowels, and his health has been rapidly failing during that time. He has a sallow skin, and a look of distress or decay, which evinces that some grave disease is gnawing like a canker at his vitals.

He had been treated by an old and skillful physician for a long time, but without improvement. He slowly, but surely, grew worse day by day.

I first saw him professionally June 14th, 1862. His condition and history impressed me with the belief that his whole trouble was gastritis. I urged upon him the importance of a firm and persevering temperance in his diet, habits, etc. Gave him a pill of rhubarb, aloes, and castile soap, *donec alvus soluta fuerit.* For irritability of stomach, he got in pill one-fourth grain nitrate of silver one hour before meals.

Did not see him until June 27th. Nitrate of silver had no sensible effect. He had taken ten of the co. rhubarb pills—two at a dose, and three times a day—also six table-spoonfuls of castor oil, all of which had produced one scanty stool of hardened fæcal lumps. Save that one small motion, there has not been, nor is not now any inclination to evacuate the bowels. His abdomen is prominent, full, and hard. Tongue is covered with a thick coating of dirty foul-looking slime. He is weak, which is as much indicated by his pulse as by his feelings. Not much thirst, no appetite. Prescribed enematas of castor oil and turpentine in starch water, to be repeated every four hours until bowels are freely moved.

June 30th.—A few scanty stools have been produced. Complained of a cutting pain in pit of stomach. Applied a small blister over epigastrium. Continued the enemas.

July 2d.—Symptoms becoming worse. Much gastric distress; vomited freely. Among the matter ejected by emesis, were pieces of onions, of which vegetable, he and his wife declared, he had not eaten for *more than two months.* After the first two or three paroxysms of vomiting, the matter was stercoraceous. On careful examination, I now discovered a tumor, firm, well defined, about four inches long, and one and a half broad, and situated vertically at the right side of, and about two inches from the umbilicus. It appeared to be situated within the abdominal cavity, was tender on pressure, and the patient never before knew that it existed. Bowels still obstinately costive. Continued injections.

6th.—Vomtting of stercoraceous matter still continues. Bowels have been opened pretty freely since my last visit. Complains of much distress in stomach. This distress is a little peculiar. He says that he has the painful impression of the contents of the bowels passing into the stomach, which, when it distends that organ, is finally got rid of by vomiting. After this emptying process, the patient has a short interval of comparative ease. There was no account taken of the quantity thus ejected, but I know that it was an almost incredible amount. It indeed seemed, by the amount of fæcal matter that came away by mouth and anum, that he had the accumulated ingesta of months to be thus got rid of. No appetite, very little thirst. Tongue loaded with slimy, rotten-looking coat; very weak. I believed that there must be mechanical obstruction of the bowels. This opinion was coincided with by my friend and neighbor, Dr. Lank, who from this date attended the case with me. However, we could not determine the nature nor seat of the obstruction. We applied a blister over the tumor.

8th.—Much worse. Vomiting of fæcal matter still continues. Debility extreme. No sleep, no appetite, great pain in the stomach. Gave him sulphate of morphia, grain one-fourth every two hours until pain abates.

10th.—Much better. Tongue cleaning, pulse fuller, distress in stomach not so great, obtained some sound sleep, has some appetite.

12th.—Not so well; skin very yellow. Bowels not moved for several days. Gave him calomel, grains two, three times a day.

13th.—Bowels moved very freely. Better.

15th.—Bowels still freely moved. The last two stools contain *very much green bile.* Remarkable improvement. What becomes of the *mechanical obstruction* now? He passes bile "fresh and green" from the liver, and that clearly proves an uninterrupted passage, at least from the duodenum downward. Has not vomited for five days.

16th.—Passed a sleepless, painful night. Took a powder of morphia, which quieted him. Felt more comfortable in the evening. Much tenderness over the stomach, where he refers most of his pain. Applied a large blister over this organ, and directed it to be made very sore.

21st.—Messenger came after me early. Found the patient suffering with severe cramp of stomach, or as he termed it, "boking." Gave him an emetic. He threw up a large quantity of water, mucus, and fæcal matter, greatly to his relief. Ordered calomel five grains at night, and injections in the morning. Also gave tincture chloride of iron, twenty drops three times a day.

23d.—Pulse 104. Countenance has a suffering, anxious look. "Boking" still continues, and is extremely painful until relieved by emesis. Great quantities of mucus discharges per anum. Profuse vomiting of dirty soily-looking fluid, which, on standing, separates into a ground-like sediment, with clear supernatent fluid. Tongue moist and little coated. Continue calomel and injections, with morphia, *ad libitum.*

24th.—Great prostration, voice weak, tongue covered with foul-looking coating. Vomits freely. Some thirst. Bowels moved. The last alvine dejections are very peculiar indeed. They consist of kidney-shaped bodies, varying in size from a common bean to a fig. They all appear to have a small pedicle or stalk. They are a bright straw color. Have much the texture and appearance of bits of fine sponge. They float lightly on water, are easily mashed between the finger and thumb into a pasty pulp, losing their spongy character. The whole amount would measure two pints. It is also proper to state that the tumor above-mentioned has shrunk wonderfully in size, although it was unreduced on the 21st. Gave him morphia every three hours. Discontinued the tincture of iron.

25th.—More comfortable. Rested tolerably well last night. This morning, passed a full and not remarkable stool. Vomited in his usual style in the forenoon, and during his last effort, while I looked on, he vomited *oil* with the matter. He had not taken oil by mouth since June 20th—more than a month ago—and he had vomited scores of times since then, yet he vomits oil! He has had several enemas of castor oil—the last two days ago—yet here he ejects oil by mouth! Pulse 98.

26th.—Passed a very large natural-looking stool without enema. Vomits profusely stercoraceous matter. Says he feels empty and miserable. Gave him sixty drops of laudanum in starch water, by injection. Was greatly relieved in the evening.

27th.—Pulse small and rapid. Stercoraceous vomiting still continues. Gave him nitrate of silver, grain one-fourth every six hours, morphia as needed for the pain, and suppostories containing each ten grains of aloes.

28th.—Sinking gradually. Suffering extreme. Vomits blood, which is intimately incorporated with mucus. Abdomen now appears flat and empty. Thirst not remarkable. Gave him laudanum injections every six hours.

29th.—Vomiting of blood, mucus, and water. The blood is intimately blended with the mucus and water, imparting to it a pale-red color.

July 31st.—He died, suffering and vomiting to the last.

*Autopsy* thirty hours after death. Present Drs. D. Donaldson and Wm. M. Miller.

The stomach contained a small quantity of bloody mucus, and also three almost worn-out cherry seed. The family assured us that he had not eaten cherries since the second week in June.

The walls of the stomach were thinner than natural, and the epithelial coat was wanting. Further than this, there was no remarkable appearance of the organ, except at the pylorus, which, with the duodenum and head of pancreas, was involved in the condition generally styled scirrhus. The parts were thickened, indurated, and gave the sensation of cartilage under the knife. The duodenum appeared to have the greatest structural change, as if the disease had its primary seat there, and had gradually encroached upon the adjacent parts. The serous coating of the gall-bladder, with all the parts of organs near it—the duodenum, colon, and serous covering of the liver were stained the color of bile, as if that secretion had permeated the walls of the gall-bladder, and spread to the neighboring tissues. The internal surface of the duodenum had a very unique appearance. The surface was covered with shallow indentations, and impressed us with the belief that the "kidney-shaped" bodies, mentioned above, had been here attached, and developed by their pedicles.

No other evidence of disease or obstruction in any other part of the intestinal canal. All other organs healthy.—*Medical and Surgical Reporter.*

